# Reduction in Salt Stress Due to the Action of Halophilic Bacteria That Promote Plant Growth in *Solanum lycopersicum*

**DOI:** 10.3390/microorganisms11112625

**Published:** 2023-10-25

**Authors:** Javier Pérez-Inocencio, Gabriel Iturriaga, Cesar L. Aguirre-Mancilla, María Soledad Vásquez-Murrieta, Marcos Alfonso Lastiri-Hernández, Dioselina Álvarez-Bernal

**Affiliations:** 1 Tecnológico Nacional de México Campus Los Reyes, Los Reyes 60330, Mexico; japeinza10@gmail.com (J.P.-I.); marcos.lastiri5@gmail.com (M.A.L.-H.); 2 Tecnológico Nacional de México Campus Roque, Celaya 38525, Mexico; gabriel.id@roque.tecnm.mx (G.I.); cesar.am@roque.tecnm.mx (C.L.A.-M.); 3 Escuela Nacional de Ciencias Biológicas, Instituto Politécnico Nacional, Mexico City 07738, Mexico; mvasquezm@ipn.mx; 4 Centro Interdisciplinario de Investigación para el Desarrollo Integral Regional Unidad Michoacán (CIIDIR-Michoacán), Instituto Politécnico Nacional, Jiquilpan 59510, Mexico

**Keywords:** plant-growth-promoting-bacteria, *Sesuvium verrucosum*, Halophyte, Halophiles

## Abstract

Soil salinity is one of the most important factors reducing agricultural productivity worldwide. Halophilic plant growth-promoting bacteria (H-PGPB) represent an alternative method of alleviating saline stress in crops of agricultural interest. In this study, the following halophilic bacteria were evaluated: *Bacillus* sp. SVHM1.1, *Halomonas* sp. SVCN6, *Halomonas* sp. SVHM8, and a consortium. They were grown under greenhouse conditions in *Solanum lycopersicum* at different salinity concentrations in irrigation water (0, 20, 60, and 100 mM NaCl) to determine the effects on germination, fruit quality, yield, and concentration of osmoprotectors in plant tissue. Our results demonstrate the influence of halophilic bacteria with the capacity to promote plant growth on the germination and development of *Solanum lycopersicum* at higher salinity levels. The germination percentage was improved at the highest concentration by the inoculated treatments (from 37 to 47%), as were the length of the radicle (30% at 20 mM) and plumule of the germinated seed, this bacterium also increased the weight of the plumule (97% at 100 mM). They also improved the yield. The dry weight of the plant, in addition to having an influence on the quality of the fruit and the concentration of osmoprotectors (*Bacillus* sp. SVHM 1.1) had the greatest effect on fruit yield (1.5 kg/plant at 20 mM), by the otherhand, *Halomonas* sp. SVHM8 provided the best fruit quality characteristics at 100 mM. According to the above results, the efficiency of halophilic PGPB in the attenuation of salt stress in *Solanum lycopersicum* has been proven.

## 1. Introduction

Tomato (*Solanum lycopersicum*) is the second-most important horticultural product in the world [1]. Being a glycophyte plant, it is moderately sensitive to salts, with a threshold of 2.5 dS/m of electrical conductivity (measured using saturation extract paste) [2]. Salinity is the most widespread and damaging abiotic factor worldwide that reduces the yield of this crop [3] due to its effects on the physiology, morphology, and biochemistry of the plant [2].

The chemical properties of some soils, such as salinity and sodicity, are conditions that restrict agricultural production [4]. The accumulation of toxic ions, reduced growth, loss of turgor in the cell, effects on photosynthesis, and stomatal conductance are just some of the problems caused by high salt concentrations [5,6], and can lead to the death of the plant [5].

Traditional breeding and genetic engineering have been used to create resistant plants and to increase the productivity of glycophyte crops [7]. Additionally, plant growth-promoting bacteria have been used as sustainable alternatives for stress attenuation, improving plants’ performance and productivity [8].

One of the less-explored options for the bioremediation of saline soils is the use of halophilic microorganisms, which have the capacity to withstand high salt concentrations as well as properties that favor plant growth. The halophilic microorganism, which can withstand high salt concentrations, are bacteria that can be found in saline environments [9]. According to their ability to survive, they are classified as halotolerant and halophilic. Halotolerant organisms are those that can tolerate high concentrations of salinity but whose conditions are not optimal, while halophilic microorganisms are those that experience growth in salinity conditions [10]. They are classified as non-halophiles: <1% NaCl (*w*/*v*); light halophiles: 1–3% (*w*/*v*) NaCl; moderately halophilic: 3–15% NaCl (*w*/*v*); and extreme halophiles: >15% NaCl (*w*/*v*) until saturation [11]. It has also been shown that these bacteria can exhibit traits that promote plant growth [12]. Halophilic plant growth-promoting bacteria (H-PGPV) are bacteria that live in the rhizosphere and promote plant growth through direct and indirect mechanisms. These include the activation of defense antioxidants by regulating enzymes such as superoxide dismutase, peroxidase, and catalase, which remove reactive oxygen species [9]; improving plant nutrition through atmospheric nitrogen fixation by solubilizing nutrients such as P and K or producing siderophores [13,14,15,16]; reducing the accumulation of Na+ in the plant by producing exopolysaccharides [17]; producing activity of the ACC deaminase enzyme, which reduces the ethylene levels of the plant; altering the structure and morphology of the roots to facilitate the absorption of nutrients; accumulating amino acids such as glutamate and proline, quaternary ammonium amines such as carnitine and glycine betaine, and sucrose and trehalose, which reduce intracellular osmotic pressure; increasing stomatal conductance and photosynthetic activity; and inducing and regulating the stress-responsive gene expression in the plant [9,17,18], thus promoting plant growth [19] and resistance to environmental stress, which ultimately increases crop yields [20].

The bacterial genera with the capacity to increase the productivity of different crops under saline stress are *Pseudomonas*, *Bacillus*, *Enterobacter*, *Agrobacterium*, *Streptomyces*, *Klebsiella*, and *Ochromobacter* [21,22,23], which demonstrate resistance to salinities of 4 to 8% NaCl. Some of these genera are resistant to levels of up to 10–15% NaCl [24]. Numerous studies have used microorganisms with plant-promoting capacities, which are found in the rhizospheres of halophytic plants, to reduce the impact of salt stress on economically significant crops [25,26,27]. Plant growth-promoting bacteria have also been widely reported for growth and yield improvement in tomato cultivation [28], with some of the reported bacteria being *Azospirillus* sp. [29], *Pseudomonas* sp. [30,31,32], *Bacillus* sp. [32], and *Erwinia* sp. [30]. These influence the improvement of root and shoot weight [29,31] and increase the lengths of root hairs, the production of hormones [29], the absorption of minerals [31], the fresh and dry weight [30], the plant height [32], and overall performance [33].

Rhizosphere bacteria of halophyte plants are effective in promoting plant growth and reducing salt stress in glycophyte crops; Halophilic bacteria of the genus *Halomonas* from halophytic plants have been rarely reported. Therefore, the objective of this work was to evaluate halophilic bacteria that promote plant growth and their effects on the germination, development, and production of *Solanum lycopersicum* at different salinity levels. It was hypothesized that inoculation with halophilic plant growth promoting bacteria (H-PGPB) in *Solanum lycopersicum* under high salinity conditions (100 mM NaCl) significantly improves germination, development, yield and fruit quality, as well as the concentration of osmoprotectants in plant tissues compared to non-inoculated plants.

## 2. Materials and Methods

### 2.1. Selection of Microorganisms

The bacteria used for this research were obtained from the bacterial bank of the Michoacán Regional Interdisciplinary Research Center for Integral Development (CIIDIR-IPN). They were characterized and identified as *Bacillus* sp. SVHM1.1, *Halomonas* sp. SVCN6, and *Halomonas* sp. SVHM8, with GenBank accession numbers ON571722, ON571727, and ON571735, respectively. These bacteria were obtained from the soil of the rhizosphere of *Sesuvium verrucosum* Raf, and possessed phosphorus solubilization capacity, indole acetic acid production (IAA) occurred through the tryptophan-dependent and -independent pathways, and ammonium was produced from an organic source; the sodium capture capacity, in addition to supporting salinity levels of 0–15% (*Bacillus* sp. SVHM1.1) and 0–20% (*Halomonas* sp. SVCN6, and *Halomonas* sp. SVHM8) with optimal growth at 5–10% in nutrient agar, and resistance to growth up to a pH of 11 [12].

### 2.2. Preparation of the Inocula

The strains selected for bioassays were incubated separately in 50 mL of nutrient broth (1 g/L meat extract; 2 g/L yeast extract; 5 g/L Peptone) [34] supplemented with 10% NaCl for 7 days at 28 °C with continuous shaking at 130 oscillations per minute. This continued until a concentration of approximately 1 Abs (absorbance) at 625 nm was reached, which is equivalent to 1.5 × 10^9^ ufc/mL [35]. For the bacterial consortium, an inoculum was prepared using a 1:1:1 ratio of *Bacillus* sp. SVHM1.1, *Halomonas* sp. SVCN6, and *Halomonas* sp. SVHM8.

### 2.3. Promotion of Plant Growth with Germination of Solanum lycopersicum under Saline Stress

In this study, *Solanum lycopersicum* seeds saladette type obtained from Hortaflor produced by Rancho Los Molinos in Mexico. They were sterilized with 70% ethanol for one minute, after which the ethanol was eliminated and they were disinfected with 1% sodium hypochlorite, then shaken at 130 rpm for 10 min [23]. Finally, six washes with distilled water were performed to remove any excess hypochlorite.

Seeds were inoculated with each of the bacteria used in this analysis by adding 10 milliliters of the inoculum that had already been adjusted for absorbance. Next, the seeds were shaken for 60 min at 130 rpm and dried at room temperature, and their viability was examined. The seeds were placed in a Petri dish with agar–water medium at different salinity levels (0, 20, 60, and 100 mM NaCl) and the following treatments were used: Control, *Bacillus* sp. SVHM1.1, *Halomonas* sp. SVCN6, *Halomonas* sp. SVHM8, and bacterial consortium. A total of 25 seeds for each treatment were incubated at 25 °C, with 16 h of light and 8 h of darkness, in a germination chamber. The experiment was performed in triplicate. The samples were observed daily until the germination percentage stopped changing from day to day (for this trial, this occurred at 12 days) [36] to determine the efficacy of the treatments.

### 2.4. Greenhouse Bioassay

*Solanum lycopersicum* seeds were germinated in peat moss substrate in a 200-cavity germination tray. After 40 days, the seedlings were transplanted into black polyethylene bags with a capacity of 5 L (12 × 12 in with gusset) with holes at a height of 2 in from the base (containing peat as a substrate). The experiment was carried out under greenhouse conditions with five repetitions. A factorial design based on the completely randomized block arrangement was used. The evaluated treatments were Control, *Bacillus* sp. SVHM1.1, *Halomonas* sp. SVCN6, *Halomonas* sp. SVHM8, and bacterial consortium. All treatments had different salinity levels in the irrigation water (0, 20, 60, or 100 mM NaCl), and were irrigated every three days. On the intermediate day, they were irrigated with Steiner nutrient solution at 2 dS/m and a pH of 6, in a ratio of 35:45:20 for K^+^:Ca^2+^:Mg^2+^ and 60:5:35 for NO_3_^-^:H_2_PO_4_^-^:SO_4_^−2^ [37]. The isolates were inoculated at the beginning of the experiment, as well as once a week until the end of the experiment, at a concentration of absorbance of approximately 1 Abs at 625 nm. The experiment lasted for a period of 180 days, starting from the transplant. During this period and at the end of it, the variables were assessed to determine the effectiveness of the bacterial inoculation.

### 2.5. Variable Analysis

For the germination test, the germination percentage, plumule emergence percentage, radicle and plumule length, radicle fresh weight, plumule fresh weight, total fresh weight, and plant vigor index, were determined by the following equation

(1) $$VI=TL\;\left(\frac{G}{10}\right)$$

where, *VI* is the vigor index, *G* is the percentage of germination and *TL* the total length of the plant [36].

In the greenhouse experiment, the variables which were evaluated to determine the effectiveness of the treatments were the fruit yield, and dry weight of the roots and the plant’s aerial part; fruit firmness; concentrations of dissolved salts in the juice of the fruit; pH; electrical conductivity; °Brix; and titratable acidity.

During the experiment, tomato fruits were harvested when they reached physiological maturity. The harvest period began 90 days after establishment and continued up to 180 days. The yield was determined by counting and weighing all the fruits from the beginning of the harvest to the end of it.

At the end of the experiment, the aerial part and root were separated. Each of the fresh parts was weighed on a 0.01 g precision analytical balance (Sartorius Group, Edgewood, Harford, MD, USA) to obtain the fresh weight of both the aerial part and the root. Subsequently, they were placed in an oven at 70 °C until a constant weight was achieved, and they were weighed to obtain the dry weight.

At 120 days after the establishment of the experiment, 30 fruits were taken at random for each treatment. The firmness of the fruit was determined with an FT20 penetrometer by puncturing the equatorial and polar part of the fruit and averaging the result. The pH, electrical conductivity, and total dissolved salts of the fresh tomato juice were determined using the HANNA HI5522-01 multiparameter (Hanna Instruments Inc., Smithfield, VA, USA). The total soluble solids (°Brix) were determined using a portable refractometer (Extech Instruments Corporation, Waltham, MA, USA) with an accuracy of ±0.2 °Brix. The titratable acidity was evaluated by the methodology described by Benito-Bautista et al. [38]; a 5-g sample of the pulp was taken and suspended in 50 mL of distilled water, adding three to five drops of 0.1% phenolphthalein.

(2) $$\%\;Citric\;Acid=\frac{V\times N\times 0.064}{Valic}*100$$

where *V* is the spent volume of NaOH, *N* is the normality of NaOH, and Valic is the volume of the aliquot.

### 2.6. Determination of the Concentration of Osmoprotectors

The proline content in plant tissue was performed according to the method described by Irigoyen et al. [39]. The leaves were frozen at 0 °C; then, 0.2 g of leaves were crushed in a mortar with 5 mL of 96% ethanol and washed with 10 mL of 70% ethanol. The samples were centrifuged for 10 min at 12,000× *g* before 2 mL of the supernatant was taken and 2.5 mL of ninhydrin reagent and 2.5 mL of glacial acetic acid were added. They were then incubated for 45 min at 100 °C. Once the samples reached room temperature, 5 mL of benzene was added. Finally, the absorbance of the samples was measured at 515 nm using a Biotek PowerWave XS2 microplate reader (Biotek Instruments, Inc., Winooski, VT, USA).

To determine the glycine betaine (GB) concentration, we used the method proposed by Grieve et al. [40]. Plant tissue extracts were obtained by shaking with H_2_SO_4_ (2N) and cooling. The extracts were mixed with periodate at a 1:1 ratio, and the samples were shaken and kept at 4 °C for 16 h. The mixture was centrifuged at 7100× *g* for 15 min at 4 °C, and the supernatant was discarded. The settled periodate crystals were dissolved in 10 mL of 1, 2-dichloroethane, then stirred and left at room temperature for 15–20 min. Finally, the absorbance at 365 nm was measured using a Biotek PowerWave XS2 microplate reader (Biotek Instruments, Inc., Winooski, VT, USA).

The trehalose content was determined by the method described by Schulze et al. [41]. First, a 0.1 g leaf sample was taken, and 2 mL of 20 mM phosphate buffer (PBS, pH 5.8) was added to homogenize the mixture. The samples were shaken in a cell shaker (Ninbo Xin Yi Science Instrument Ltd., Co., Ninbo, Zhejiang) for 30 min. They were centrifuged at 10,300× *g* for 10 min at 4 °C before 200 μL of the supernatant was taken and mixed with 200 μL of 1% H_2_SO_4_. The samples were then placed in a water bath at 90 °C for 10 min. Then, they were cooled on ice for approximately 3 min, 200 μL of 30% NaOH was added, the supernatant was recovered, and 3 mL of developer agent (0.02 g of anthrone (Sigma, St. Louis, MO, USA)) was added. The samples were placed into a water bath again at 90 °C for 10 min. They were cooled on ice for 3 min; then, 10 mL of 80% H_2_SO^4-^ was added. Finally, the absorbance at 630 nm was measured using a Biotek PowerWave XS2 microplate reader (Biotek Instruments, Inc., Winooski, VT, USA).

### 2.7. Statistical Analysis

The data obtained from the variables that were evaluated were averaged and submitted to the Shapiro-Wilk normality test (*p* ≤ 0.05) and the Levene variance homogeneity test. The evaluation of the germination was carried out in triplicate, and was analyzed by two-way ANOVA and Tukey’s test (*p* ≤ 0.05). For the greenhouse bioassay, five plants per treatment were used, and each treatment was subjected to four salinity levels. Variables were analyzed using two-way ANOVA and Tukey’s test (*p* ≤ 0.05) to determine significant mean differences, Minitab^®^ statistical software was used (version 17 for Windows).

## 3. Results

The evaluation of the effect of bacterial inoculation on the germination of *Solanum lycopersicum* was evaluated at various salinity levels (0, 20, 60, and 100 mM NaCl).

For all of the variables evaluated in the germination test, an adverse effect was observed when the concentration of NaCl was increased, with the physiological impact caused by saline stress being evident (Table 1 and Table 2). The statistical analysis shows significant differences between the treatments and the different levels of salinity, where the bacterial inoculation, specifically *Bacillus* sp. SVHM1.1, presents a greater effect at salinity levels of 20 and 60 mM compared to the control. Although germination was negatively affected by a salinity of 100 mM in all treatments, the control treatment did not exceed 33% germination; while the bacterial inoculation improved seed germination from 36 to 47%, being *Halomonas* sp. SVHM8 the one with the highest percentage of germination; the control treatment did not exceed 33% germination; at this level of salinity, the bacterial consortium did not have the desired effect (Table 1).

The percentage of emergence of plumules was negatively affected by the increase in salinity concentration. At 0 mM NaCl, the average of the treatments was close to 24% plumule emergence, while at 100 mM, the percentage varied by 1%. However, each level of salinity evidenced the beneficial influence of bacterial inoculation on the length of the radicle, with values statistically higher than those of the control; the two-way statistical analysis shows a greater influence by *Halomonas* sp. SVHM8 (24.0 ± 0.6). *Bacillus* sp. SVHM1.1 presenting the greatest effect on radicle length, at a salinity of 20 mM (11.3 ± 0.8) presenting a significant difference (*p* ≤ 0.05). The effect of bacterial inoculation was more evident for the length of the plumule, the bacterial consortium had the greatest influence with 4.8 ± 0.3 (at 0 mM).

Regarding the radicle weight, the control treatment presented greater weight compared to the inoculated treatments, for 100 mM all of the treatments presented negative effects with a weight reduction of between 54% and 66% respect al control a 0 mM. For the plumule weight, the treatment with the greatest influence was the bacterial consortium (0 and 20 mM salinity) with 310 ± 11.0 and 299.0 ± 14.4 mg, respectively; at 100 mM, positive effect of the bacterial inoculation became more evident, with that of *Halomonas* sp. SVCN6 being 97% statistically greater than the control at that salinity. For the total fresh weight, *Halomonas* sp. SVCN6 presented a higher percentage of effect (100 Mm of NaCl) compared to the control, with a 73% greater fresh weight; *Bacillus* sp. SVHM1.1 (0 mM) and the consortium (20 mM) had a greater influence on this variable according to the statistical analysis. Finally, for the seedling vigor variable, the treatment that presented the greatest influence was *Halomonas* sp. SVHM8 con 13% greater vigor respect al control a 0 mM and 438% greater vigor compared to the control of 100 mM.

The germination variables such as percentage of plumule, plumule length, plumule weight, total weight and germination rate were positively stimulated by the inoculation of bacteria of the genus *Halomonas* and the consortium, demonstrating the effectiveness of the bacteria in raising the value of the variables evaluated; and with greater effect at levels of 20 and 60 mM NaCl.

### 3.1. Greenhouse Evaluation

#### 3.1.1. Fruit Yield

The results clearly show the effect of salinity in each of the treatments. It is evident that by increasing the salinity concentration, the production per plant was directly decreased (Table 3 and Table 4). For the salinity level of 0 mM NaCl, the influence of bacterial inoculation on the yield was clearly observed. The treatment of *Bacillus* sp. SVHM1.1 had the greatest effect at 20 mM salinity, with 25% higher yield compared to the control at 0 mM and 34% higher than the control at 20 mM, followed by the *Halomonas* sp. SVHM8, with a 21% higher yield a 20 mM, which compared to the control treatment at 0 mM. By increasing the salinity, bacterial inoculation has a greater influence on increasing plant yield; *Halomonas* sp. SVHM6 and *Bacillus* sp. SVHM1.1 at 100 mM NaCl presented statistically significant differences (*p* ≤ 0.05) respect to control, increasing productivity by 56% and 50%, respectively.

#### 3.1.2. Dry Weight of the Plant

The influence of the inoculation of the bacterial isolate on the growth of the dry plants was, once again, notable; there were significant differences (*p* ≤ 0.05) between the treatments (Table 3 and Table 4). The dry weight, *Halomonas* sp. SVHM8 statistically outperformed the other treatments with 211 ± 24.7 g/plant at 20 mM salinity.

#### 3.1.3. Fruit Quality

The results of the variables evaluated to determine the quality of the fruit varied, showed significant differences (*p* ≤ 0.05) (Table 5). An increase in salinity had an effect on the decrease in pulp pH. In the case of red tomato, a pH below 4.5 indicates good quality [42], all treatments are below this level. *Bacillus* sp. SVHM1.1 (60 mM of salinity), *Halomonas* sp. SVCN6 and consortium (at 100 mM salinity) had the lowest pH, this below the rest of the treatments at different salinities. Although it is within the acceptable level of quality, the control treatment was the one with the highest pH with 8% above those that registered the lowest pH.

The salinity had an effect on the total dissolved salts in the fruit; increasing the concentration increased along with the level of salinity. Once again, the inoculated treatments presented higher concentrations of dissolved salts a 100 mM de NaCl, *Halomonas* sp. SVHM8 and the consortium, which stood out in approximately 13% compared to the control at the same salinity, and a 38% higher concentration compared to the control treatment at 0 mM. The percentage of citric acid in pulp was also affected by the increase in NaCl concentration, as well as by bacterial inoculation; at 100 mM NaCl the greatest effect was seen by the *Halomonas* sp. SVHM8 treatment, presented higher percentages of citric acid with 42% more than the lowest concentration (control at 0 mM). For the total sugar, at each level of salinity, the inoculated treatments presented significant differences (*p* ≤ 0.05) with respect to the control treatment.

*Halomonas* sp. SVHM8 presented a higher concentration of brix degrees at 100 mM NaCl, con 16% higher concentrations than the control treatment at the same salinity and 51% higher than the control treatment at 0 mM (which was the one with the lowest sugar concentration). As for fruit firmness, the treatments show significant differences (*p* ≤ 0.05); at 100 mM NaCl, *Halomonas* sp. SVHM8 presented a firmness 22% better than that of the control treatment (100 mM NaCl) and 38% greater than control treatment at 0 mM.

The inoculation of the H-PGPB bacteria improves the growth of the crop when subjected to saline stress, increasing the dry weight of the plant at the three levels of salinity, increasing the yield, as well as the quality of the fruit, compared to non-H-PGPB bacteria inoculated (with a greater effect on those isolated from the genus *Halomonas*).

### 3.2. Osmoprotectors

Proline, trehalose, and glycine betaine were the quantified osmoprotectors whose concentrations in the plant tissue were evaluated 180 days after the culture was established. Bacteria of the *Halomonas* genus (with greater influence *Halomonas* sp. SVHM8) had a greater effect on the concentration of osmoprotectants at 20 and 60 mM salinity, as described below.

The proline concentration was determined in each of the treatments, and they were statistically compared to determine the greatest effect at different salinity levels (Table 6). For 0 mM NaCl, the bacterial consortium treatment presented a 35% increase in proline concentration compared to the control treatment at the same salinity and 79% above the treatment with less influence (control at 20 mM), while at 20 mM, the *Halomonas* sp. SVHM8 had a concentration 83% higher than that of the control a 20 mM; for 60 mM, again *Halomonas* sp. SVHM8, which presented a 24% higher concentration compared to the control at the same salinity and 42% higher concentration compared to the one with less influence (control at 20 mM). The trehalose levels in the leaf tissue decreased as the salinity increased. At 20 mM, the treatment inoculated with *Halomonas* sp. SVCN6, statistically presented the highest concentration; it is interesting to observe a reduction of this osmorpotector by this same bacterium at 60 mM salinity, with reductions of 68% compared to this same inoculum at 20 mM. The glycine betaine concentration presented significant differences (*p* ≤ 0.05) between the treatments at each level of salinity. In addition, a reduction was observed in the glycine betaine concentration of the control treatment (except 0 mM NaCl). Comparing these results, the influence of the bacterial consortium increases in the concentration of this osmoprotectant at 20 mM NaCl, with 1.7 mM/g glycine betaine in plant tissue.

## 4. Discussion

In this study, the influence of halophilic bacteria with the capacity to promote plant growth on the germination and development of S. lycopersicum at different salinity levels was evidenced. In some treatments, inoculation improved the percentage of germination at the highest salt concentration, and improved the radicle and plumule lengths in the germinated seeds. It also increased the weight of the plumules, and the fresh weights of the germinated seeds. The yield and the dry weight of the plant were also improved, and there was an influence on the quality of the fruit and the concentration of osmoprotectors against saline stress in the plant. Numerous studies have used microorganisms found in the rhizospheres of halophytic plants with the ability to promote plant growth to reduce the impact of salt stress on economically significant crops. The inoculation of bacteria on *S. lycopersicum* under saline stress had a greater influence with respect to the control treatment at the different salinity levels. These results are in agreement with previous reports on the evaluation of halophilic bacteria that promote plant growth in tomato under saline stress, showing an improvement in germination, an increase in seedling vigor, increased root and stem length, higher dry weight of the plant, and increased concentration of proline in the leaves compared to those not inoculated, by bacteria such as *Glutamycybacter halophytcola* [43], *Bacillus velenzensis*, and *Bacillus subtilis* subsp. spizizenii [44], bacterial consortium formed by Achromobacters sp., *Bacillus* sp., *Bacillus sonorensis*, *Delftia* sp. and *Enterobacter* sp. [45]; *Bacillus aryabhattai* and *B. mesonae* [46]; *Arthrobatcer* and *Bacillus metaterium* [47]; and *Azotobacter chroococcum* [4,5], to name a few. Halophilic bacteria that promote plant growth have been evaluated in cultures of interest, and the results indicate the importance of bacterial inoculation for managing the stress to which the plant is subjected [9,48,49,50]. The results of inoculated alfalfa seeds with bacteria isolated from halophyte plants showed that grew faster than the seeds of the control treatment in the presence of salinity. Sharma et al. [21] examined the role of bacteria isolated from the halophyte *Arthrocneum indicum* in peanut germination under salt stress, and found that the inoculated treatments exhibited better growth. Sanchez Lopez et al. [25] evaluated the effect of re-inoculated halophilic bacteria from the rhizosphere of *Pennisetum clandestum*, under saline stress conditions, increased the plant height, root length, and the dry weight of the aerial part and the root of the plant with respect to the control at high salinity levels. Farhangi-Abriz et al. [25] used Pseudomonas RS 198 and *Azospirillum brasilense* RS SP7 as plant growth promoters in *Brassica napu* at three levels of salinity, showing results in terms of reducing the effect of salinity on the plant and increasing the levels of phosphorus, calcium, and magnesium; a greater leaf area, greater plant biomass, higher levels of chlorophyll a and b, and an improvement in photosynthetic efficiency were also achieved. Sultana et al. [27] isolated and characterized plant growth-promoting bacteria from saline soils and evaluated them in an Oryza sativa culture at various salinity levels, the bacteria used were *Ochrobactrum intermedium*, *Achromobacter denitrificans*, and *Bacillus megaterium*, these bacteria increase the length of the crop, the chlorophyll content, the protein content, the carbohydrate content, and the dry weight in inoculated plants. The beneficial effect of inoculation by H-PGPB and the attenuation of saline stress is due to its ability to absorb salts, in addition to its ability to promote growth by influencing its metabolic activities, enzyme production, solubilization of soil nutrients, production of phytohormones, and ability to adapt to pH and salinity stress [12,19,20].

Another response of plants to saline stress is the accumulation of osmolytes, which increases when subjected to abiotic stress due to its role in the osmotic adjustment of the cell, helping plants to survive this effect [51]. The solutes compatible with the plant are varied, with some of them being proline, trehalose, and glycyl betahine [52,53]. Proline production can act on reactive oxygen species, leading to the regulation of cytosolic acidity. The stabilization of proteins [54] is also related to the combined actions of enzymes of most bacteria which are needed to withstand stress [55]. The results of this research put the bacteria as those with the greatest effect in the increase of this compound, being able to be part of the responsible metabolism in the reduction of saline stress to which the crop was subjected [9,18]. Trehalose is an osmoprotectant and halophilic bacteria play an important role in its production in plants. Bacteria produce it in order to survive stress, and at the same time, they can provide the plant with enzymes necessary for the production of the trehalose [55]. For this investigation, the bacterial consortium enhanced the concentration of trehalose in plant tissue to 60 mM, while *Halomonas* sp. SVCN6 in 20 mM NaCl. *Glycine betaine* is another osmoprotector that is associated with increased salt stress accumulating in the cytosol; it thereby maintains cell integrity [56]. Most of the works reporting on the inoculation of halophilic bacteria and its effect on the production of osmoprotectors in cultures of interest subjected to saline stress agree that the bacteria increase the concentration of these osmoprotectors [16,57,58]. It is interesting to analyze in particular the maximum salinity concentration (100 mM NaCl), where the bacterial inoculation reduced the production of proline and trehalose, while glycine betaine increased. The inoculation of plant growth-promoting bacteria can influence the increase or decrease in the production of osmoprotectors at high salinity levels; considering that this reduction in the production of osmoprotectors is evidence of the adaptation of the inoculated plant to stress [59]. This can be attributed to the beneficial effect of bacteria on the reduction in reactive oxygen species and in Na^+^ concentration in the rhizosphere, production of enzymes and the increase in nutrient solubility finally reduced the stress in the rhizospheres of the plants, which was reflected in the reduction of the expression of some osmoprotectors in the plant.

## 5. Conclusions

In the present investigation, various strains of halophilic plant growth-promoting bacteria (H-PGPB) were evaluated under high salinity conditions for the cultivation of *Solanum lycopersicum*. The results obtained reveal that the introduction of these bacteria had a notable impact on various aspects of the crop; specifically, there was an improvement in seed germination, seedling development, yield and fruit quality, along with an elevation in the concentration of osmoprotectants in plant tissues.

Data analysis indicated that *Bacillus* sp. SVHM 1.1 had the most significant effect on fruit yield, while *Halomonas* sp. SVHM8 improved fruit quality under high salinity conditions. These findings corroborate the effectiveness of H-PGPBs in mitigating salt stress in *Solanum lycopersicum*, suggesting that this approach could be a promising alternative to address soil salinity and increase crop production in regions affected by this situation, furthermore, this study demonstrates the potential of halophilic plant growth-promoting bacteria as a valuable tool for agriculture in regions with saline soils, which could contribute to food security and agricultural sustainability worldwide.

## Figures and Tables

**Table 1:** Effect of the inoculation of bacteria with characteristics of growth promotion on the germination and length of *Solanum lycopersicum*.

Salinity (mM NaCl)	Treatments	Germination %				Plumule Emergence %				Radicle Length (cm)				Plumule Length (cm)				Radicle Weight (mg)				Plumule Weight (mg)				Total Weight (mg)				Seedling Vigor			
0	Control	92	±	0.0	ab	22	±	0.6	ab	10.3	±	0.6	ac	4.0	±	0.1	ad	136.3	±	13.1	a	261.0	±	7.0	ad	397.3	±	16.2	ab	132.2	±	5.8	ad
	*Bacillus* sp. SVHM1.1	92	±	0.0	ab	22	±	1.0	ab	9.0	±	1.0	bf	4.3	±	0.4	ab	126.7	±	10.4	ac	283.3	±	15.3	ab	410.0	±	22.9	a	122.7	±	11.0	af
	*Halomonas* sp. SVCN6	96	±	4.0	a	22	±	2.1	ab	7.2	±	0.8	eh	3.8	±	0.3	be	104.3	±	2.5	df	274.7	±	5.5	ad	379.0	±	7.8	ac	105.5	±	12.7	df
	*Halomonas* sp. SVHM8	99	±	2.3	a	24	±	0.6	a	11.0	±	1.0	ab	4.1	±	0.2	ac	131.0	±	4.6	ab	233.7	±	11.6	bd	364.7	±	10.4	ac	149.1	±	11.4	a
	Consortium	95	±	6.1	a	23	±	1.2	ab	7.5	±	0.5	dh	4.8	±	0.3	a	90.0	±	8.0	f	310.7	±	11.0	a	400.7	±	19.0	ab	116.0	±	4.6	bf
20	Control	93	±	1.0	ab	15	±	2.6	cd	8.7	±	0.8	cg	3.7	±	0.3	bf	114.3	±	9.0	be	271.0	±	10.1	ad	385.3	±	15.4	ac	115.6	±	7.5	bf
	*Bacillus* sp. SVHM1.1	95	±	2.3	a	18	±	0.0	bc	11.3	±	0.8	a	3.3	±	0.3	df	97.3	±	7.1	ef	222.3	±	28.6	cd	319.7	±	29.3	c	138.3	±	9.8	ac
	*Halomonas* sp. SVCN6	95	±	2.3	a	16	±	1.5	cd	11.0	±	1.0	ab	3.8	±	0.3	be	114.7	±	2.5	ae	221.0	±	16.5	d	335.7	±	18.6	bc	140.4	±	12.2	ab
	*Halomonas* sp. SVHM8	92	±	6.4	ab	18	±	2.3	bc	9.3	±	1.0	ae	3.4	±	0.3	cf	120.0	±	10.0	ad	233.0	±	35.6	bd	353.0	±	39.0	ac	117.5	±	13.6	bf
	Consortium	95	±	2.3	a	19	±	2.3	ac	7.2	±	0.5	eh	4.5	±	0.3	ab	107.7	±	2.5	cf	299.0	±	14.4	a	406.7	±	16.4	a	111.1	±	7.6	cf
60	Control	91	±	2.3	ab	12	±	2.1	d	10.3	±	0.8	ac	3.1	±	0.1	ef	118.0	±	6.2	ae	272.7	±	12.2	ad	390.7	±	18.0	ab	122.2	±	9.6	af
	*Bacillus* sp. SVHM1.1	93	±	2.3	a	14	±	1.5	cd	10.9	±	0.6	ab	3.0	±	0.5	fg	122.7	±	6.7	ad	272.0	±	15.1	ad	394.7	±	15.3	ab	130.0	±	7.2	ae
	*Halomonas* sp. SVCN6	80	±	4.0	b	12	±	1.7	d	9.6	±	0.9	ad	3.3	±	0.2	cf	101.0	±	4.4	df	267.3	±	29.7	ad	368.3	±	25.4	ac	103.5	±	4.4	ef
	*Halomonas* sp. SVHM8	89	±	4.6	ab	15	±	1.5	cd	7.9	±	0.4	dg	3.3	±	0.3	df	113.0	±	9.8	be	260.7	±	34.0	ad	373.7	±	41.8	ac	100.2	±	10.7	f
	Consortium	91	±	4.6	ab	14	±	2.3	cd	10.7	±	0.6	ac	3.7	±	0.3	bf	106.0	±	2.6	cf	280.0	±	17.7	ad	386.0	±	20.1	ac	131.3	±	12.6	ad
100	Control	33	±	2.3	d	2	±	1.5	e	6.7	±	0.3	gi	1.6	±	0.2	h	46.7	±	1.5	g	72.7	±	6.4	f	119.3	±	6.7	e	27.7	±	2.1	g
	*Bacillus* sp. SVHM1.1	37	±	5.0	cd	2	±	1.2	e	5.4	±	0.6	hi	1.6	±	0.1	h	53.7	±	11.2	g	86.3	±	18.5	ef	140.0	±	28.2	de	26.0	±	6.1	g
	*Halomonas* sp. SVCN6	36	±	6.9	cd	1	±	1.0	e	6.9	±	0.5	fh	1.7	±	0.3	h	63.0	±	1.0	g	143.3	±	17.5	e	206.3	±	16.5	d	31.0	±	5.5	g
	*Halomonas* sp. SVHM8	47	±	8.3	c	1	±	1.0	e	4.6	±	0.8	i	1.7	±	0.2	h	43.7	±	4.0	g	86.3	±	14.4	ef	130.0	±	10.4	e	29.2	±	4.3	g
	Consortium	30	±	5.5	d	1	±	0.6	e	5.5	±	0.2	hi	2.3	±	0.3	gh	49.3	±	4.5	g	127.7	±	19.8	ef	177.0	±	21.5	de	22.9	±	3.8	g

The statistical comparison of the treatments was by two-way ANOVA and Tukey’s test (*p* ≤ 0.05). The values indicate the average ± standard error of the replicates evaluated, different letters at each salinity level indicate significant differences (*p* ≤ 0.05).

**Table 2:** Effects of the factors analyzed from the two-way ANOVA and the significant interactions between the factors (Treatments, Salinity and Treatments × Salinity) on the germination variables.

	Germination %	Plumule Emergence %	Radicle Length (cm)	Plumule Length (cm)	Radicle Weight (mg)	Plumule Weight (mg)	Total Weight (mg)	Seedling Vigor
*F*-Value								
Treatment	2.8 *	2.46	8.09 ***	17.41 ***	9.35 ***	11.89 ***	4.62 **	2.16
Salinity	643.51 ***	436.62 ***	101.97 ***	240.77 ***	297.3 ***	272.53 ***	394.85 ***	438.99 ***
Treatment × Salinity	3.16 **	1.14	13.08 ***	1.69	8.98 ***	4.23 ***	4.81 ***	8.16 ***

* *p* ≤ 0.05; ** *p* ≤ 0.01; *** *p* ≤ 0.001.

**Table 3:** Effect of bacterial inoculation on the dry weight and yield of *Solanum lycopersicum*.

Salinity (mM NaCl)	Treatments	Fruit Yield (g/Planta)				Fresh Weight of Plant (g)				Dry Weight of Plant (g)			
0	Control	1126	±	36.0	ce	431	±	50.8	j	162	±	20.1	ad
	*Bacillus* sp. SVHM1.1	1107	±	67.0	df	620	±	43.0	hj	162	±	5.6	ad
	*Halomonas* sp. SVCN6	1264	±	44.5	bd	842	±	79.5	dh	175	±	27.2	ad
	*Halomonas* sp. SVHM8	1337	±	111.0	ac	523	±	90.7	ij	151	±	15.6	bd
	Consortium	1136	±	30.0	ce	845	±	103.4	dh	147	±	24.0	bd
20	Control	995	±	49.0	eh	969	±	85.7	ce	182	±	19.9	ad
	*Bacillus* sp. SVHM1.1	1520	±	28.5	a	945	±	80.3	cg	158	±	9.5	ad
	*Halomonas* sp. SVCN6	939	±	64.0	ei	1144	±	25.7	ac	139	±	11.5	bd
	*Halomonas* sp. SVHM8	1443	±	80.0	ab	1062	±	102.6	ad	211	±	24.7	a
	Consortium	1086	±	58.5	dg	689	±	116.2	gj	155	±	14.6	ad
60	Control	848	±	34.0	hi	892	±	57.4	cg	140	±	25.7	bd
	*Bacillus* sp. SVHM1.1	977	±	60.5	eh	922	±	71.8	cg	127	±	12.9	d
	*Halomonas* sp. SVCN6	898	±	78.1	fi	784	±	98.3	eh	175	±	14.7	ad
	*Halomonas* sp. SVHM8	1127	±	128.3	ce	700	±	113.5	fi	149	±	24.6	bd
	Consortium	874	±	44.5	gi	1238	±	90.2	ab	194	±	20.8	ab
100	Control	552	±	46.5	j	1107	±	64.9	ac	170	±	22.5	ad
	*Bacillus* sp. SVHM1.1	831	±	76.0	hi	1266	±	78.7	a	177	±	4.2	ad
	*Halomonas* sp. SVCN6	860	±	56.0	hi	960	±	100.1	cf	185	±	17.0	ac
	*Halomonas* sp. SVHM8	595	±	69.0	j	1143	±	85.5	ac	131	±	20.4	cd
	Consortium	726	±	107.6	ij	1006	±	80.6	be	157	±	19.3	ad

The statistical comparison of the treatments was by two-way ANOVA and Tukey’s test (*p* ≤ 0.05). The values indicate the mean ± standard error of the replicates evaluated, different letters at each salinity level indicate significant differences (*p* ≤ 0.05).

**Table 4:** Effects of the factors analyzed from the two-way ANOVA and the significant interactions between the factors (Treatments, Salinity and Treatments × Salinity) on the fruit yield, and dry weight of plant.

	Fruit Yield (g/Planta)	Fresh Weight of Plant (g)	Dry Weight of Plant (g)
*F*-Value			
Treatment	27.35 ***	3.72 *	0.73
Salinity	169.88 ***	73.23 ***	1.22
Treatment × Salinity	14.69 ***	15.86 ***	5.63 ***

* *p* ≤ 0.05; *** *p* ≤ 0.001.

**Table 5:** Effect of bacterial inoculation on the quality of the *Solanum lycopersicum* fruit.

Salinity (mM NaCl)	Treatments	pH				Total Dissolved Salts (g/mL)				Total Dissolved Sugar (°Brix)				Firmness (lbf)				Citric Acid (%)			
0	Control	4.22	±	0.04	a	3.17	±	0.06	fh	4.7	±	0.1	i	9.2	±	0.8	d	0.44	±	0.02	h
	*Bacillus* sp. SVHM1.1	4.10	±	0.03	bd	2.89	±	0.04	h	4.7	±	0.1	i	10.4	±	1.0	ad	0.44	±	0.02	h
	*Halomonas* sp. SVCN6	4.03	±	0.01	cg	3.12	±	0.19	gh	6.7	±	0.1	ab	11.9	±	1.1	ac	0.63	±	0.02	cd
	*Halomonas* sp. SVHM8	4.11	±	0.02	bc	3.14	±	0.15	gh	5.0	±	0.2	i	9.9	±	1.0	bd	0.44	±	0.02	h
	Consortium	3.99	±	0.01	eh	3.20	±	0.22	fh	6.9	±	0.1	ab	9.5	±	1.3	cd	0.60	±	0.02	cd
20	Control	4.13	±	0.01	b	3.42	±	0.11	eg	5.1	±	0.1	i	10.8	±	0.8	ad	0.45	±	0.02	gh
	*Bacillus* sp. SVHM1.1	3.98	±	0.06	eh	3.63	±	0.18	bf	5.5	±	0.3	gh	10.8	±	0.8	ad	0.45	±	0.02	gh
	*Halomonas* sp. SVCN6	4.05	±	0.01	be	3.54	±	0.12	cg	4.9	±	0.1	i	10.6	±	0.5	ad	0.52	±	0.02	ef
	*Halomonas* sp. SVHM8	3.94	±	0.04	gh	4.07	±	0.21	ab	6.6	±	0.2	bc	10.6	±	0.4	ad	0.70	±	0.02	ab
	Consortium	3.99	±	0.02	eh	3.45	±	0.07	dg	5.1	±	0.1	i	9.1	±	0.4	d	0.47	±	0.02	fh
60	Control	4.02	±	0.02	dg	3.89	±	0.20	bd	5.1	±	0.1	hi	11.2	±	1.0	ad	0.52	±	0.04	ef
	*Bacillus* sp. SVHM1.1	3.92	±	0.03	h	3.82	±	0.21	be	6.5	±	0.1	bd	12.3	±	0.6	ab	0.63	±	0.02	cd
	*Halomonas* sp. SVCN6	4.06	±	0.04	be	3.82	±	0.12	be	5.7	±	0.1	fg	9.9	±	1.1	bd	0.56	±	0.02	de
	*Halomonas* sp. SVHM8	4.03	±	0.04	cf	3.70	±	0.23	be	4.9	±	0.1	i	11.1	±	0.9	ad	0.51	±	0.02	eg
	Consortium	4.04	±	0.03	cf	3.99	±	0.07	ac	5.7	±	0.1	fg	10.3	±	0.8	ad	0.56	±	0.02	de
100	Control	3.99	±	0.02	eh	3.86	±	0.16	be	6.1	±	0.1	df	10.4	±	0.5	ad	0.52	±	0.02	ef
	*Bacillus* sp. SVHM1.1	4.03	±	0.04	cf	3.71	±	0.19	be	5.9	±	0.1	eg	11.7	±	0.6	ad	0.52	±	0.02	ef
	*Halomonas* sp. SVCN6	3.91	±	0.06	h	3.63	±	0.07	bf	5.7	±	0.1	g	12.2	±	0.8	ab	0.56	±	0.02	de
	*Halomonas* sp. SVHM8	3.95	±	0.03	fh	4.37	±	0.11	a	7.1	±	0.1	a	12.7	±	1.2	a	0.77	±	0.04	a
	Consortium	3.91	±	0.01	h	4.38	±	0.09	a	6.2	±	0.2	ce	9.9	±	0.7	bd	0.67	±	0.02	bc

g/mL = grams per milliliters; lbf = pound force; % = percentage. The statistical comparison of the treatments was by two-way ANOVA and Tukey’s test (*p* ≤ 0.05). The values indicate the mean ± standard error of the replicates evaluated, different letters at each salinity level indicate significant differences (*p* ≤ 0.05).

**Table 6:** Effect of bacterial inoculation on the concentration of osmoprotectors in leaf tissue of *Solanum lycopersicum*.

Salinity (mM NaCl)	Treatments	Proline (mM/g)				Trehalose (mM/g)				Glycine Betaine (mM/g)			
0	Control	0.818	±	0.085	bc	27.87	±	0.72	bc	1.53	±	0.19	ab
	*Bacillus* sp. SVHM1.1	0.866	±	0.008	b	27.41	±	0.66	bc	1.38	±	0.15	ae
	*Halomonas* sp. SVCN6	0.783	±	0.022	bc	27.67	±	2.85	bc	1.04	±	0.13	df
	*Halomonas* sp. SVHM8	0.880	±	0.054	b	27.75	±	1.38	bc	1.28	±	0.01	af
	Consortium	1.107	±	0.036	a	25.99	±	2.47	cd	1.41	±	0.18	ae
20	Control	0.617	±	0.095	d	25.41	±	0.26	cd	1.23	±	0.17	bf
	*Bacillus* sp. SVHM1.1	0.730	±	0.105	bd	19.63	±	1.00	ef	1.00	±	0.01	ef
	*Halomonas* sp. SVCN6	0.852	±	0.001	b	34.53	±	2.35	a	1.48	±	0.09	ac
	*Halomonas* sp. SVHM8	1.129	±	0.006	a	20.14	±	0.54	ef	1.21	±	0.15	bf
	Consortium	0.785	±	0.045	bc	27.36	±	1.80	bc	1.70	±	0.27	a
60	Control	0.863	±	0.004	b	32.25	±	2.82	ab	0.86	±	0.08	f
	*Bacillus* sp. SVHM1.1	0.764	±	0.032	bd	16.67	±	0.72	fh	1.05	±	0.06	cf
	*Halomonas* sp. SVCN6	0.822	±	0.003	bc	12.87	±	0.69	h	0.94	±	0.07	f
	*Halomonas* sp. SVHM8	1.072	±	0.002	a	32.23	±	1.96	ab	1.14	±	0.07	bf
	Consortium	0.780	±	0.026	bc	33.82	±	3.16	a	1.14	±	0.09	bf
100	Control	0.872	±	0.000	b	22.99	±	0.96	ce	0.99	±	0.05	ef
	*Bacillus* sp. SVHM1.1	0.796	±	0.045	bc	18.55	±	0.18	eg	1.29	±	0.14	af
	*Halomonas* sp. SVCN6	0.842	±	0.006	b	14.52	±	0.58	gh	1.26	±	0.16	bf
	*Halomonas* sp. SVHM8	0.674	±	0.035	cd	16.55	±	0.22	fh	1.14	±	0.18	bf
	Consortium	0.753	±	0.096	bd	21.71	±	0.62	de	1.43	±	0.18	ad

mM = millimoles; g = grams of leaf tissue. The statistical comparison of the treatments was by two-way ANOVA and Tukey’s test (*p* ≤ 0.05). The values indicate the mean ± standard error of the replicates evaluated, different letters at each salinity level indicate significant differences (*p* ≤ 0.05).

## Data Availability

The data presented in this study are available on request from the corresponding author.
